# Dr. Sanders, on Disorders of the Nervous System

**Published:** 1818-01

**Authors:** James Sanders

**Affiliations:** 24, James Square, Edinburgh


*To the Editors of the Medico-Chirurgical Journal & Review.*

GENTLEMEN,

IN your Journal and Review for October last, I read with particular interest, Mr. Webster's Case of Hydrophobia: the treatment was judiciously conducted, and the remarks evince observation; but the account of the examination of the body after death, affords the most ample scope for reflection; here we see the relation subsisting between the signs of disease, and the changes which the organization undergoes, during its operation; this is by far the most important part of medical investigation.

The appearances detailed are precisely of the same kind with those which I have uniformly found in trismus, asthma, pertussis, croup, and all spasmodic affections, where the more remarkable effects are produced from the mouth to the diaphragm inclusively; all of them frequently prove fatal, and that suddenly, without having been accompanied with any strong marks of pyrexia. It is worthy of attention, that in those who have died of croup, whether of the species called inflammatory, or the spasmodic, effusion more or less is always found in the cerebellic cavity and at the top of the spine, as well as turgescence of the veins surrounding the medulla oblongata, and the nerves thence arising; and, for the most part, the greater the serous effusion, the less conspicuous is the turgescence. This turgid state of the vessels often involves the whole base of the brain, and extends down the cervical part of the spinal marrow; but this range of morbid action internally, is denoted during life by a corresponding extension of morbid signs; so intimately in effect are these connected, and so nicely do the signs indicate the locality, if I may so express myself, of the distending vessels on the nervous system, that here, as my friend Dr. Seeds can inform you, before opening a body, a statement was always made of what we expected to find; nor did the appearances, when the parts were exposed, ever materially differ, in extent or degree, from our anticipation.

Considering the symptoms, as detailed of Mr. Webster's patient, I should have expected the base of the brain and the continuation of the cerebrum and the cerebellum, and their nerves, to be severely affected down to the first of the dorsal vertebræ, because the parts convulsed were the face, the neck, the superior extremities, and the diaphragm, the principal nerves of which arise within the space just pointed out. The contraction of the colon would have induced me to expect, that some of the splanchaic nerves were covered with red vessels at the origins; but the total absence of convulsions in the inferior extremities, and of morbid contraction in the ilium and rectum, indicates but little disorder to have been in the dorsal, and very little or none, in the lumbar portion of the spinal marrow. With regard to the bloody serum which flowed so copiously and so long, from the vertebral canal,—this probably was from the basilar veins cut, in taking out the superior portion of the spinal cord, which are always, in such instances much enlarged; as well as from the sinus venosi so freely communicating with one another all along the osseous cavity of the spine.

When I commenced my inquiries concerning the relations which might exist between the phænomena of disease and the changes which simultaneously occur in the nervous system, I was led into so many mistakes by judging of one part from the appearances in another, that I resolved never to proceed in description, beyond the limits of the parts exposed; and adhering to this rule, we gradually obtained that mass of facts, which enabled us to discern, with such exactness, the influence exerted by certain changes in the brain, spinal marrow, and their nerves, conjointly and individually, on the organs subservient to them; consequently, when the functions of the organs were disordered, we had a definite indication of what was going on in the nervous system.

The modes of treatment which these anatomical researches have suggested, at length begin to be pretty generally, though imperfectly, adopted; indeed, for the utility, as well as the honour of our profession, it is desirable that there were more of understanding, and less of imitation in the adoption; hands and judgement should be employed in ascertaining what is done, and ought to be done, rather than ears and memory in collecting vague rumours concerning something done, by persons here and elsewhere. Instead of saying, " that of late, several practitioners in this city have begun to examine the state of the spinal marrow in diseases, and that the same thing is also done by certain gentlemen on the Continent, which will, no doubt, be useful in illustrating many affections, in which this part of the nervous system is chiefly concerned :" And, instead of saying, that " the contents of the spine may be easily exposed by turning the body on the face, with pillows of straw under it, and then by dissecting away the muscles from the spinous processes, and cutting off these processes with a saw :" After a little information to be easily acquired, it would have been stated, that much valuable knowledge had been obtained relative to the structure and functions of the nervous system, in health and disease; and, that it had been accomplished in this city, by the labour of years, and in defiance of every obstacle ; in defiance of smuggled dissections of the most important cases; and in defiance of all the obloquy of malevolence, and all the hostility of confederate ignorance.

It would have been stated, that in Britain, as well as on the Continent, in dissecting rooms, the contents of the spine were sometimes exposed by opening the cavity from behind; but, that this method was not adapted to the purposes of morbid anatomy, since it could not be followed in private houses, where alone the ravages of disease could be seen a few hours after death, and before putrefaction had done material injury; that in consequence, the structure of the spinal marrow, the relative size, situation, and connection of its parts, the origin of its nerves, the circulation and distribution of its vessels, and the manner in which they are involved during disease, all remain a mystery, except to those who have seen the operation performed in a much more expeditious way a short time after death; and where the symptoms, during the progress of the respective maladies, had been carefully observed; for when the symptoms of the disease have not been attended to, the examination of the cadaver may lead to pernicious error, and never can be but of inferior utility.

No opportunity has been neglected for many years, of confirming or increasing the number of our observations by the use of the scalpel, and repetition suggested many facilities. We had long explored the whole encephalon, and the spine in general partially, and had hence perceived, that the symptoms of certain diseases were invariably connected with the morbid changes occurring in these parts respectively. Instructed by the comparison of accumulated facts, I affirmed in my Lectures, that in all those cases on record, where anatomists had examined bodies, and declared that nothing could be discovered to account for the fatal event, if they had opened the spine they would have been undeceived; that, in short, all of them were instances in which the seat of the disease was within the spinal canal.

One day, when I made this remark, some of the gentlemen attending my course heard of a child who had died of a short illness: the cavities of the head, thorax, and abdomen, had been inspected without affording the slightest appearances to explain the death. They went and asked permission to open the spine, and sent me notice that they had obtained it. When I arrived, I found my ingenious friend, Dr. John Smith, opening the spinal canal, by sawing through the bodies of the vertebræ in the interior, or, as Dr. Barclay would more correctly say, at the sternal aspect. He soon exposed the contents, unhurt by the operation: on this occasion we had one decisive proof of what I had stated, for the spinal marrow, its nerves and envelopes, were diseased from the atlas to the sacrum; the vessels covered by the arachnoid coat were turgid; the dura mater was of a dark crimson colour, and surrounded with coagulated lymph; serum filled the canal; the sinus venosi were dark and distended.

This happened several years ago; from which time we have, in the majority of cases, opened the head and spine completely; nor do we ever overlook the viscera of all the other cavities.

The more bodies I examine, the more am I astonished how little of precision has been acquired in the knowledge of the nervous system, and of its blood-vessels, as well as of their co-relative action in all the phænomena of living beings. The sum-total of a calculation in arithmetic does not more depend on the value of the digits, than the functions of animals do on the mutual influence incessantly, while life remains, exerted between the nervous and vascular systems. Their conjoint action, duly proportioned throughout the body, constitutes health; to maintain or restore the equilibrium constitutes the whole art of medicine.

Those who are not intimately acquainted with the mechanism, can neither regulate nor correct the machine; if ever their attempts seem successful, it depends on fortuitous occurrences, which they can neither detect nor appreciate. Thus nervous maladies, and some believed to be of a different nature, after having been pronounced incurable, have vanished spontaneously, as if to hold in derision the boast of skill and the pride of science. But the whole is vanity, when the science is without facts, and the skill without knowledge.

The labours of Drs. Gall and Spurzheim enable anatomists to have a more accurate idea of the distribution and arrangement maintained among the parts composing the brain, cerebellum, and the continuations of both to the basilar extremities of the pyramidal eminences; but it is much to be regretted, that their scalpel had not explored the spinal cord below the decussation with equal dexterity and perseverance: not that their anatomy of the encephalon is yet complete, though it certainly approaches nearer perfection than that of the spinal elongation; and it is astonishing, since their object was the explanation of the functions of the brain, that they made no observations on the co operation of the vascular system, in all the actions and exertions both of the centre of the nervous system, and of the nerves from their origins to their ulterior destinations. Still, however, they deserve the thanks and esteem of every friend of science, for having surpassed all their predecessors and contemporaries in this department.

When we read, in anatomical works, that the spinal marrow consists of four columns, the posterior the smaller; that there are two lateral grooves, and that the four columns can be separated from each other, all their length, except at the decussation, or some other place not so well defined, we should believe, that these things had been seen, and could be demonstrated : but what are we to suppose, when, on examination, no such grooves are to be found, and seldom any appearances admitting of such description, except, perhaps, at the sides of the corpora olivaria, where we find, that the continuations from the cerebellum are nothing more than two triangular slips, indented, so to speak, between the posterior edges of the crura cerebri, and both not occupying one-sixth of the circumference? when, in fine, we find that the longitudinal portions of the spinal marrow, so far from being separable without lesion of structure, are, their whole length, as completely united by medullary matter at their apices towards the centre, as the two sides of the brain are by the corpus callosum?

Some have described the spinal marrow as composed of fasciculi of medullary fibres placed longitudinally, increased by the accession of nervous filaments; but, by a little careful dissection, we ascertain, that the striated or nervous appearance in the longitudinal direction, is scarcely perceptible below the pyramidal bodies, but, on the contrary, from the decussation to the termination of the cord, the striæ all proceed horizontally from the medullary connection at the centre, and are prolonged into the nervous filaments composing the spinal nerves; and further, that the blood-vessels pass between the columns of the cord, in the same horizontal direction, conducted by the pia mater assuming the form of a ligamentum dentatum; and minute vessels are seen passing through small puncta in the above-mentioned central union.

There was published, a few days ago, in a Medical Journal, the case of a patient who died of an injury received on the spine, with the appearances on dissection; in which it is stated, that the medullary and cineritious substances, at a certain part, were so blended, that the "*central brown column*" of the spinal cord, could not be distinguished. Medical men might hence infer, that a fifth column had been discovered. The spinal marrow, however, has no greater claim to five columns, than a quadruped has to five feet; nor did the cineritious substance, or brown matter if you will, ever assume any thing like the columnar form.

Every one must agree with you, that such cases as that of Mr. Webster, "let in a flood of light on nervous and spasmodic diseases;" and, after you have continued your labours through a series of diseases, of different type and character, you will not be surprised that death shonld occur from affections of the head, or any other part, unpreceded by the general symptoms of pyrexia. You will also be satisfied that there is *one part,* till which fail, death never occurs: that is, the medulla oblongata. No matter where the disease appears to commence, whether in the extremities or any intermediate situation; whether in the brain or spinal marrow; the nearer to *this* part the greater the danger. In all morbid affections of any consequence, this part must be, as much as in our power, protected: its functions lost, life ceases.

Tetanic, and other nervous disorders, arising from local injuries, have a diversity in their progress and phænomena corresponding with the parts injured. Tetanus occasioned by a wounded finger differs conspicuously from that occasioned by a wounded toe.

The contents of the head and spine, with the nerves there commencing, exhibit the same appearances in partial that they do in general convulsions, and the same appearances in partial that they do in general paralyses; each differing only in extent and degree, according to the symptoms: though, it ought to be remembered, that the appearances in convulsions or violent movements, voluntary or involuntary, are very different in kind from what they are in paralyses, or where motion is deficient. They are as different, indeed, as the phænomena of the two forms of disease.

The appearances also in those who have died of fever alone, are very different from what are found in those who have died of convulsions, or paralysis; and where fever has been combined with either, or both, the appearances in the cadaver are equally diversified.

The anatomical differences between typhus and inflammatory fever, are as remarkable as those between convulsions and paralysis; and the latter bears the same relation to typhus that the former does to inflammatory fever. It seems not improper here to make a remark of the greatest practical utility; that in fevers, the head is sometimes severely, sometimes slightly, and sometimes scarcely at all affected: but whether the head has suffered much or not, we have not found one instance of fever, properly so called, in which the spinal marrow and its membranes, including the medulla oblongata, have not evinced, throughout their whole length, the ravages of morbid action. In short, the spinal canal is the chief seat of every general disease, or fever; and to this, *a fortiori*, our means of cure ought to be chiefly directed.

This is so deeply impressed on my mind, from what I have seen and done, that I am convinced, if the spine were attended to in the commencement, or during that period commonly, though improperly, called the *predisposition*, fever would very seldom advance, and the instances of death from it would be still more rare. I doubt, truly, if ever fever would prove fatal, except when combined with some local affection, which previously existed, and had been making, perhaps, imperceptible progress.

Recent publications on fevers, and communications from the continent of Europe, show that our proceedings have already made an extensive impression. Though my pupils have been few, they have been active and intelligent, and attracted solely by the love of the subject, one of whom is worth a million of timid " groundlings." But efforts made to deter students from attending my lectures, and to excite prejudices in the mind of the public against our pursuits, only render us more determined and persevering. It gives me much pleasure, indeed, to observe that the participators in these investigations, have neither been inept, idle, nor indolent: they have brought the subject before societies, and before the world, in journals; they have carried these ideas with them to every part of the globe. Truth is too powerful for opposition : the interests of selfish individuals must yield to those of humanity. How infinitely preferable is a reputation founded on knowledge which time cannot shake, to wealth rapidly acquired at the expence of the credulity and lives of our fellow mortals !

It was not my intention to commence, at present, any systematic details : this I will do, with your permission, through the medium of your publication, as soon as time will allow. I have merely proposed to give, in a desultory manner, such memoranda as might assist those who wish to unite their efforts with ours, in establishing the practice of medicine on the solid basis of anatomy.

Sincerely wishing prosperity to your independent and spirited Journal,

I am, &c. &c.

JAMES SANDERS.

24, *James Square, Edinburgh,*

*Nov.* 1817.

